# Context-Dependent Modulation of Epithelial Barrier Integrity and Intestinal Permeability by Transcutaneous Auricular Vagus Nerve Stimulation in Two Preclinical Models Mimicking Ulcerative Colitis and Crohn’s Disease: A Descriptive Analysis

**DOI:** 10.3390/ijms27146109

**Published:** 2026-07-08

**Authors:** Fatemeh Hesampour, Olivia Johnson, Charles N. Bernstein, Jean-Eric Ghia

**Affiliations:** 1 Department of Immunology, Max Rady College of Medicine, Rady Faculty of Health Sciences, University of Manitoba, Winnipeg, MB R3E 0W3, Canada; hesampof@myumanitoba.ca (F.H.); johns380@myumanitoba.ca (O.J.); 2 Paul Albrechtsen Research Institute, CancerCare Manitoba, Winnipeg, MB R3E 0V9, Canada; 3 Department of Internal Medicine, Max Rady College of Medicine, Rady Faculty of Health Sciences, University of Manitoba, Winnipeg, MB R3E 0J9, Canada; charles.bernstein@umanitoba.ca; 4 Inflammatory Bowel Disease Clinical & Research Centre, University of Manitoba, Winnipeg, MB R3A 1R9, Canada; 5 Children’s Hospital Research Institute of Manitoba, Winnipeg, MB R3E 3P4, Canada

**Keywords:** colitis, epithelial cells, mucosal barrier integrity, non-invasive vagus nerve stimulation, stem cells, transcutaneous auricular vagus nerve stimulation, vagus nerve stimulation

## Abstract

Inflammatory bowel disease, including ulcerative colitis (UC) and Crohn’s disease (CD), is associated with reduced vagus nerve activity and impaired barrier function. Transcutaneous auricular vagus nerve stimulation (taVNS) shows preventive effects in acute colitis, but its impact on intestinal barrier integrity remains unclear. To assess this, C57BL/6 male mice received taVNS (10 V, 20 Hz, 500 µs, 10 min) prior to colitis induction. UC-like colitis was induced using 5% dextran sulfate sodium (DSS) for 120 h with daily taVNS, while CD-like colitis was induced by intrarectal dinitrobenzene sulfonic acid (DNBS, 4 mg) in 30% ethanol with taVNS during induction and for 48 h. TaVNS reduced colonic proliferation in non-colitic mice in a homeostatic manner, as well as in DNBS-colitic mice, and enhanced differentiation. It decreased enteroendocrine cells in non-colitic conditions but not in DSS-colitic mice, and reduced tuft cells in DSS and DNBS groups. TaVNS increased MUC2 in DSS colitis and decreased TFF3 in DNBS controls. It decreased Lgr5^+^ stem cells in DSS controls but maintained them during DSS colitis, while HOPX^+^ stem cells decreased in non-colitic DSS conditions, and fetal-like stem cells remained unchanged. Disease activity index negatively correlated with chromogranin A. TaVNS prevented increased paracellular permeability in the distal colon of DSS-colitic mice, increased TEER in distal DSS controls and proximal colitic colon, and enhanced ion transport in the proximal colon under non-colitic DSS conditions. Overall, taVNS effects on epithelial composition and barrier function are context- and model-dependent.

## 1. Introduction

Inflammatory bowel disease (IBD) includes two main subtypes: ulcerative colitis (UC) and Crohn’s disease (CD). The exact cause of these inflammatory conditions, which affect about 0.7 percent of Canadians, remains unclear [1,2]. However, they have been linked to impaired epithelial barrier integrity, increased intestinal permeability, and intestinal dysbiosis [3]. Prior studies have shown increased ulcerative lesions and mucosal damage, as well as disruptions in various functions of colonic epithelial cells (CECs), including proliferation, differentiation, and mucus production [3]. The intestinal mucosa is lined by a thin columnar epithelium that serves as a physical and biochemical barrier, maintaining gut homeostasis in steady states. Several types of CECs differentiate from standard epithelial stem cells to form a single-cell layer covering the intestinal lining [2]. Three key types of intestinal stem cells include proliferating leucine-rich repeat-containing G protein-coupled receptor 5 (Lgr5)-positive stem cells, lymphocyte antigen 6 A (Ly6A)-positive fetal-like stem cells, and homeodomain-only protein homeobox (HOPX)-positive quiescent stem cells. These stem cells aid in tissue regeneration and epithelial differentiation in the gut [4]. They give rise to enterocytes, enteroendocrine cells (EECs), goblet cells, and tuft cells. Any disruption in the differentiation, proliferation, or function of these cells can lead to mucosal changes associated with IBD, such as ulcerative lesions and increased permeability [2]. The pathogenesis of IBD is also linked to diminished parasympathetic nervous system (PaNS) activity, particularly involving the vagus nerve (VN), as seen in some IBD patients [5]. The VN plays a role in regulating various gastrointestinal (GI) functions, including permeability, motility, secretion, and immunity [6]. Given the VN’s crucial role, several preclinical and clinical studies have reported the anti-inflammatory effects of invasive vagus nerve stimulation (VNS) and non-invasive transcutaneous auricular VNS (taVNS), showing promising results in IBD treatment [7,8,9,10,11]. In both settings, activation of brainstem nuclei following VNS involves the nucleus tractus solitarii (NTS) in the medulla and the dorsal motor nucleus of the VN (DMV), which are the two primary activated nuclei, with the DMV containing efferent fibers of the VN that innervate the GI tract [12,13]. However, innervation density by the VN varies across different regions of the GI tract. For instance, in humans, the VN directly innervates the esophagus, stomach, small intestine, and the proximal half of the transverse colon, but not the left colon or rectum. Additionally, VN innervation patterns are species-specific. In mice, the VN densely innervates the cecum and proximal colon, with less innervation in the mid and distal colon, whereas humans lack innervation of the distal colon [14,15].

Part of the VN’s role in regulating GI functions may involve direct and indirect interactions between vagal fibers and different subsets of CECs and stem cells [16,17]. Direct interactions occur between vagal fibers and certain epithelial cells, such as tuft cells and EECs [16,17]. Tuft cells are chemosensory cells that detect chemicals, such as neurotransmitters produced by vagal fibers, and contribute to tissue regeneration [2,17]. Some EECs form glutamatergic synapses with vagal afferents in the small intestine and distal colon [18]. The VN is also vital for regulating mucus secretion and goblet cell functions in the GI tract [19,20,21]. Moreover, because these cells interact with and are in proximity to stem cells at the crypt bottoms, it is believed that vagal fibers may indirectly influence stem cells [22].

In our previous study [7], we demonstrated the model-dependent preventive effects of taVNS on colitis development using two preclinical models of colitis: the UC-like dextran sulfate sodium (DSS)-induced acute colitis and the CD-like dinitrobenzene sulfonic acid (DNBS)-induced colitis mouse models. However, in the present descriptive study, we investigated the potential preventive effects of taVNS on intestinal proliferation, epithelial differentiation, intestinal stem cells, differentiated CECs, and overall barrier function using the same mouse models.

## 2. Results

### 2.1. The Effects of taVNS on the Colonic Proliferative and Differentiated Cells in the DSS-Induced Colitis Model

**Figure 1:**
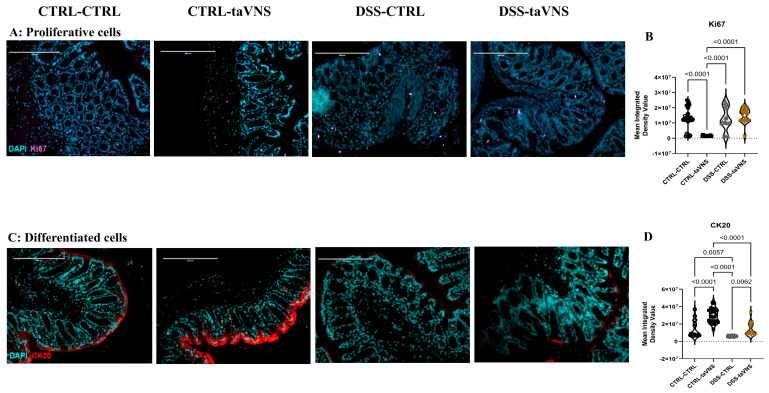
The effects of transcutaneous auricular vagus nerve stimulation (taVNS) on the proliferative and differentiated cells in the distal colon of a dextran sulfate sodium (DSS)-induced acute colitis model. Twenty-four hours before the induction of acute colitis, the taVNS and sham (CTRL) groups received taVNS stimulation condition (frequency: 20 Hz, pulse width: 500 µs, duration: 10 min with 30-s on/off intervals) or sham stimulation condition (anesthesia with no stimulation), respectively. The same taVNS/sham stimulation procedure was followed daily until the end of the experiment. Twenty-four hours later, acute colitis was induced in the DSS groups by adding 5% DSS to their drinking water for 120 h, with control (non-colitic) groups continuing to receive drinking water without any DSS. The mean integrated density (MID) of the following parameters was evaluated in the distal colon using immunofluorescence (IF) staining: the proliferation marker Ki67 (**A**,**B**) and differentiation marker CK20 (**C**,**D**). The groups were statistically compared using one-way ANOVA, followed by a multiple parametric comparison test (Tukey’s HSD post hoc). *p*-values < 0.05 were considered statistically significant. Data were presented as mean ± SD. The scale bar was 200 μm. Each group consisted of n = 4 mice.

Although taVNS significantly reduced the mean integrated density (MID) of the proliferation marker Ki67 in non-colitic mice, there were no significant modifications in this marker in colitic mice (Figure 1A,B). TaVNS increased the MID of the differentiation marker CK20 in non-colitic conditions. Additionally, taVNS prevented the colitis-induced decrease in the MID of CK20 in DSS-taVNS mice (Figure 1C,D).

### 2.2. The Effects of taVNS on the Differentiated Epithelial Cells: Enteroendocrine-, Tuft-, and Goblet Cell-Associated Markers in the DSS-Induced Colitis Model

**Figure 2:**
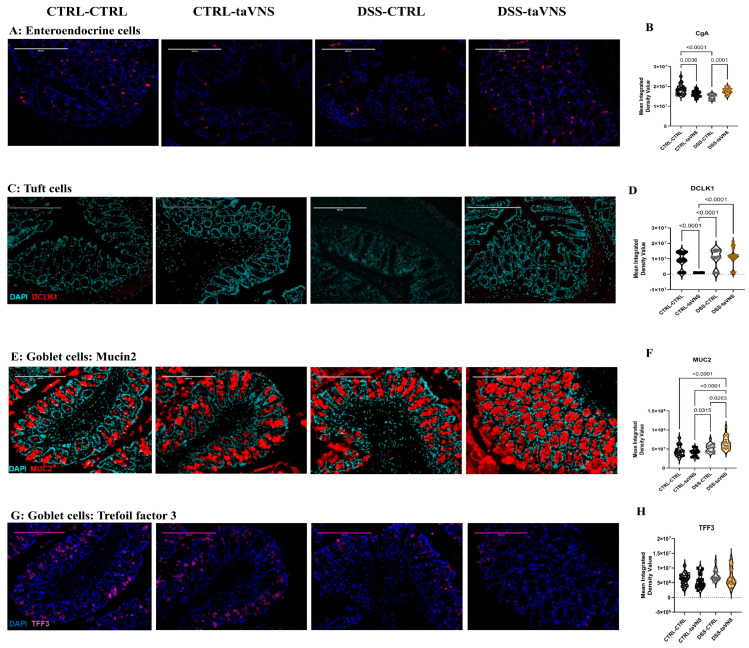
The effects of transcutaneous auricular vagus nerve stimulation (taVNS) on enteroendocrine cells, tuft cells, and goblet cells in the distal colon of a dextran sulfate sodium (DSS)-induced acute colitis model. The mean integrated density (MID) of the following parameters was evaluated in the distal colon using immunofluorescence (IF) staining: the enteroendocrine-cell-related chromogranin A (CgA) (**A**,**B**), tuft cell-associated doublecortin-like kinase 1 (DCLK1) (**C**,**D**), goblet cell-associated markers, including mucin2 (MUC2) (**E**,**F**) and trefoil factor 3 (Tff3) (**G**,**H**). The groups were compared using one-way ANOVA, followed by a multiple parametric comparison test (Tukey’s HSD post hoc). *p*-values < 0.05 were considered statistically significant. Data were presented as mean ± SD. The scale bar was 200 μm. Each group in IF experiments consisted of n = 4 mice.

TaVNS significantly decreased the MID of CgA (Figure 2A,B) in non-colitic mice and prevented the colitis-induced decrease in the MID of this marker in DSS-taVNS mice (Figure 2A,B). TaVNS significantly decreased the MID of DCLK1 marker in non-colitic mice but did not modify it in colitic conditions (Figure 2C,D). No changes were observed in *Dclk1* mRNA expression under either non-colitic or colitic conditions (Supplementary Figure S1A). No significant changes in the MID of MUC2 marker were observed in non-colitic conditions; however, taVNS significantly increased the MID of this marker in colitic mice (Figure 2E,F). TaVNS did not modify *Muc2* mRNA levels across all groups (Supplementary Figure S1B). TFF3, another goblet cell-related marker, showed no significant changes in either non-colitic or colitic mice (Figure 2G,H).

### 2.3. The Effects of taVNS on the Colonic Proliferating, Fetal-like, and Quiescent Stem Cells in the DSS-Induced Colitis Model

**Figure 3:**
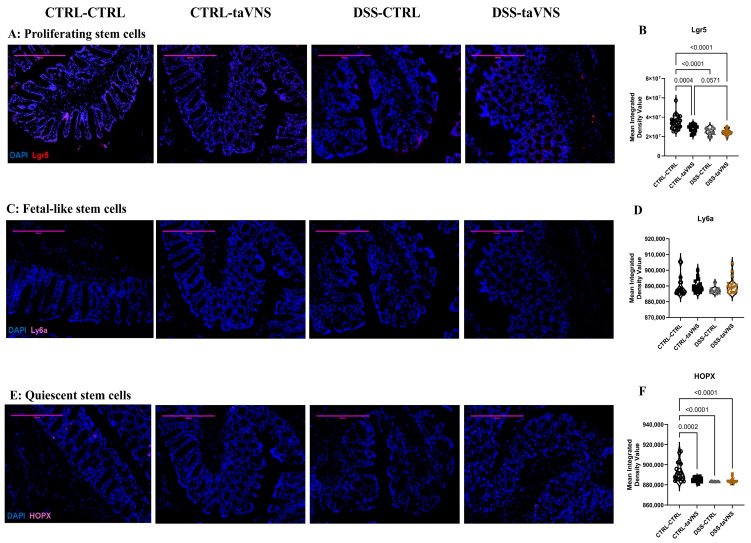
The effects of transcutaneous auricular vagus nerve stimulation (taVNS) on proliferating, fetal-like, and quiescent stem cells in the distal colon of a dextran sulfate sodium (DSS)-induced acute colitis model. The mean integrated density (MID) of the following parameters was evaluated in the distal colon using immunofluorescence (IF) staining: the proliferating-stem-cell-associated leucine-rich repeat-containing G-protein-coupled receptor 5 (Lgr5) (**A**,**B**), fetal-like-stem-cell-related lymphocyte antigen 6 A (Ly6A) (**C**,**D**), and quiescent stem cell marker homeodomain-only protein homeobox (HOPX) (**E**,**F**). The groups were statistically compared using one-way ANOVA followed by a multiple parametric comparison test (Tukey’s HSD post hoc). *p*-values < 0.05 were considered statistically significant. Data were presented as mean ± SD. The scale bar was 200 μm. Each group in IF experiments consisted of n = 4 mice.

TaVNS decreased the MID of Lgr5 in non-colitic mice (Figure 3A,B). Despite a significant decrease in the MID of Lgr5 in colitic mice, taVNS did not affect this marker in colitic conditions (Figure 3A,B). TaVNS decreased colonic mRNA expression of *Lgr5* in non-colitic mice and prevented a significant decrease in the mRNA expression levels of this marker in colitic mice (Supplementary Figure S2A). No significant differences were observed regarding the MID and mRNA expression levels of Ly6a, in both non-colitic and colitic mice (Figure 3C,D and Figure S2B). TaVNS significantly decreased the MID of HOPX in non-colitic mice. In colitic conditions, MID of HOPX was significantly decreased, but taVNS did not affect it (Figure 3E,F). Although the mRNA expression level of *Hopx* was significantly decreased in the DSS-CTRL group, taVNS also did not modify it (Supplementary Figure S2C).

### 2.4. Correlation Between the DAI-AUC and MID of Various Epithelial Cell-Associated Markers in the DSS-Induced Colitis Model

**Figure 4:**
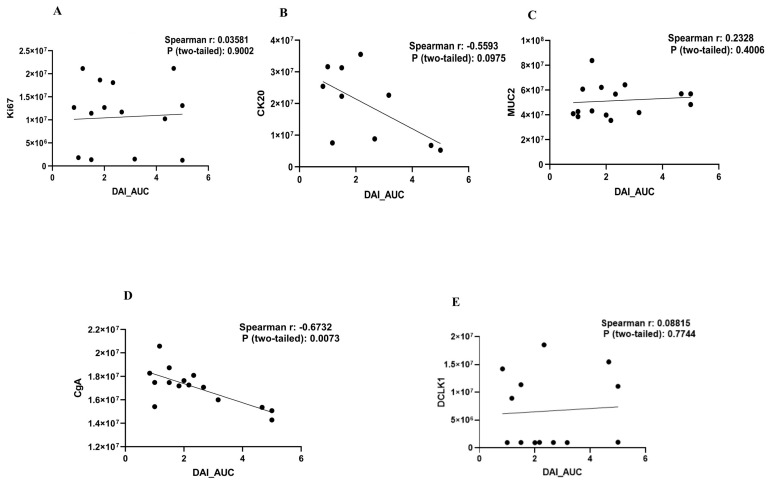
Correlation between the area under the curve (AUC) of the disease activity index (DAI) and the protein levels of differentiation and proliferation markers and gut-differentiated epithelial cell-associated markers in a dextran sulfate sodium (DSS)-induced acute colitis model. Using the data shown in Figure 1 and Figure 2 and our previously published DAI data [7], as well as the calculated AUC, the correlation between AUC-DAI and the protein levels of the following parameters was calculated for each mouse: antigen Kiel 67 (Ki67) (**A**), cytokeratin 20 (CK20) (**B**), mucin2 (MUC2) (**C**), chromogranin A (CgA) (**D**), and doublecortin-like kinase 1 (DCLK1) (**E**). Spearman’s r and corresponding *p* values were calculated using Spearman’s non-parametric test. The fitted solid line is shown for visual purposes only and was not included in the statistical analysis. Statistical significance was set at *p* < 0.05. In the correlation analysis, the data from all experimental groups were pooled, and each dot on each graph represents one mouse (3–4 mice per group, 13–16 mice total).

We examined whether there was any correlation between the evaluated epithelial cell-associated markers in the previous sections and the DAI-AUC from our previously published data [7]. There was no significant correlation between DAI-AUC and the colonic expression, quantified by MID, of Ki67 (Figure 4A), CK20 (Figure 4B), MUC2 (Figure 4C), and DCLK1 (Figure 4E). However, Spearman’s rank correlation analysis revealed a significantly strong negative association between DAI-AUC and CgA (Spearman’s r = −0.6732, *p* (two-tailed) = 0.0073) (Figure 4D).

### 2.5. The Effects of taVNS on Paracellular Permeability, TEER, as Well as the Epithelial Transport Activity in Response to Different Drugs in the Distal and Proximal Colon

**Figure 5:**
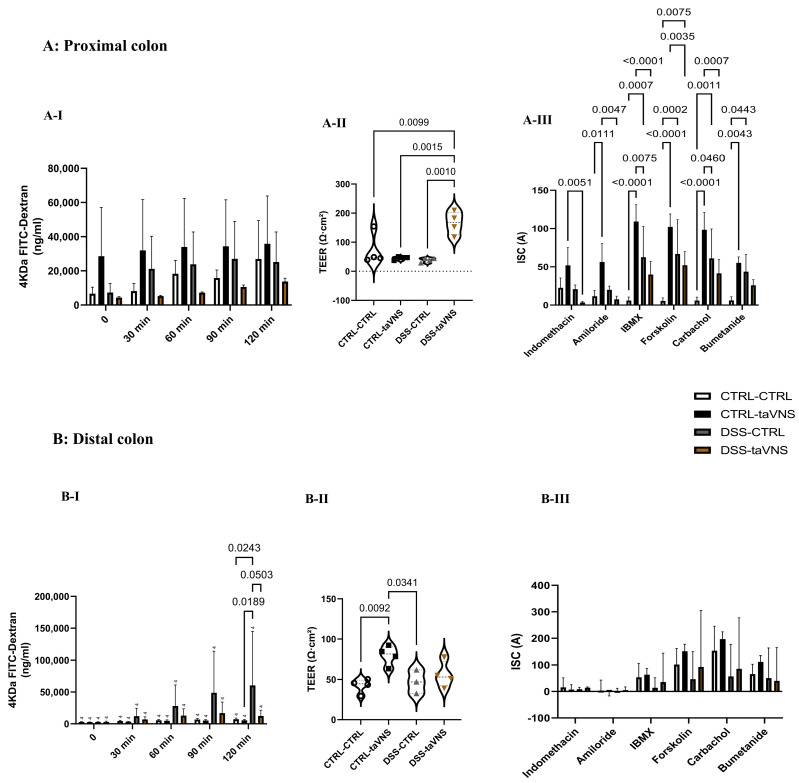
The impact of transcutaneous auricular vagus nerve stimulation (taVNS) on paracellular permeability, transepithelial electrical resistance (TEER), as well as the epithelial transport activity in response to various drugs in the distal and proximal colon using a dextran sulfate sodium (DSS)-induced acute colitis model. A similar protocol was followed, as described in the caption of Figure 1. Using the collected tissues from the distal and proximal colon, the following parameters were analyzed in the Ussing chamber: paracellular permeability, determined with FITC-dextran 4 kDa ((**A-I**) for the proximal colon and (**B-I**) for the distal colon), transepithelial electrical resistance (TEER) ((**A-II**) for the proximal colon and (**B-II**) for the distal colon), and the epithelial transport activity in response to different drugs ((**A-III**) for the proximal colon and (**B-III**) for the distal colon). The groups were compared using two-way or one-way ANOVA followed by a multiple parametric comparison test (Tukey’s HSD post hoc). *p*-values < 0.05 were considered statistically significant. Data were presented as mean ± SD. Each group consisted of n = 4 mice.

In the proximal colon, taVNS did not affect FITC-dextran flux over 120 min (Figure 5A-I). In addition, two-way ANOVA showed a significant main effect of treatment group (*p* = 0.0004), whereas the effects of time (*p* = 0.23) and the group-by-time interaction (*p* = 0.99) were not significant. TaVNS did not modify TEER in the proximal colon of non-colitic mice; however, it increased TEER in the proximal colon of the DSS-taVNS group (Figure 5A-II). TaVNS significantly increased the ion transport activity of proximal colon tissues from non-colitic mice in response to amiloride, IBMX, forskolin, carbachol, and bumetanide, with no significant difference in response to indomethacin (Figure 5A-III). However, no significant difference in responsiveness to those pharmacological agents was observed in the proximal colon of colitic mice. Additionally, two-way ANOVA demonstrated significant main effects of treatment group (*p* = <0.0001) and drug (*p* = <0.0001), as well as a significant interaction between group and drug (*p* = 0.01), indicating that the effects of the pharmacological treatments were significantly different among the experimental groups.

In the distal colon, no significant changes in FITC-dextran flux across non-colitic samples were detected (Figure 5B-I). In colitic samples, paracellular permeability of the distal colon did not change significantly over 90 min of measurement. At the 120-min time point, colitic samples demonstrated a significant increase in FITC-dextran flux, and taVNS almost significantly decreased it (*p* value = 0.0503) (Figure 5B-I). Two-way ANOVA showed a significant main effect of treatment group (*p* = 0.006), whereas the effects of time (*p* = 0.2) and the group-by-time interaction (*p* = 0.7) were not significant. In non-colitic conditions, taVNS significantly increased TEER in the distal colon (Figure 5B-II). No significant differences were observed among the colitis groups. Moreover, no significant differences in epithelial transport activity (ISC) in the distal colon were observed among the groups when tested against pharmacological agents (Figure 5B-III). In addition, two-way ANOVA revealed a significant main effect of drug (*p* = 0.0004), whereas neither the treatment group effect (*p* = 0.1) nor the drug and treatment interaction (*p* = 0.97) reached statistical significance.

### 2.6. The Effects of taVNS on the Colonic Proliferative and Differentiated Cells in the DNBS-Induced Colitis Model

**Figure 6:**
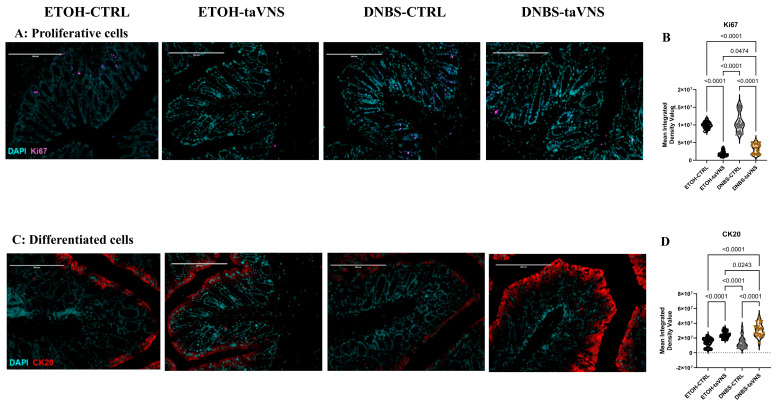
The effects of transcutaneous auricular vagus nerve stimulation (taVNS) on the proliferative and differentiated cells in the distal colon of a dinitrobenzene sulfonic acid (DNBS)-induced acute colitis model. Based on their assigned groups, twenty-four hours before receiving DNBS or 30% ethanol (ETOH) treatment, the mice were subjected to taVNS (20 Hz, 500 µs, 30-s on/off duration, and 10-min stimulation duration) or sham stimulation (anesthesia with no stimulation). These conditions were maintained daily throughout the experiment. After twenty-four hours, the DNBS groups were administered a single intrarectal injection (100 µL) of 4 mg/mouse DNBS dissolved in 30% ETOH at time point 0, whereas the ETOH groups received only 30% ethanol. The mean integrated density (MID) of the following parameters was evaluated in the distal colon using immunofluorescence (IF) staining: the proliferation marker antigen Kiel 67 (Ki67) (**A**,**B**) and differentiation marker cytokeratin 20 (CK20) (**C**,**D**). The groups were statistically compared to each other using one-way ANOVA followed by a multiple parametric comparison test (Tukey’s HSD post hoc). *p*-values < 0.05 were considered statistically significant. Data were presented as mean ± SD. The scale bar was 200 μm. Each group consisted of n = 4 mice.

In both control and DNBS-induced colitic conditions, taVNS significantly decreased the MID of the proliferation marker Ki67 (Figure 6A,B). However, taVNS increased the MID of the differentiation marker CK20 in both control and colitic mice (Figure 6C,D).

### 2.7. The Effects of taVNS on the Differentiated Epithelial Cells: Enteroendocrine-, Tuft-, and Goblet Cell-Associated Markers in the DNBS-Induced Colitis Model

**Figure 7:**
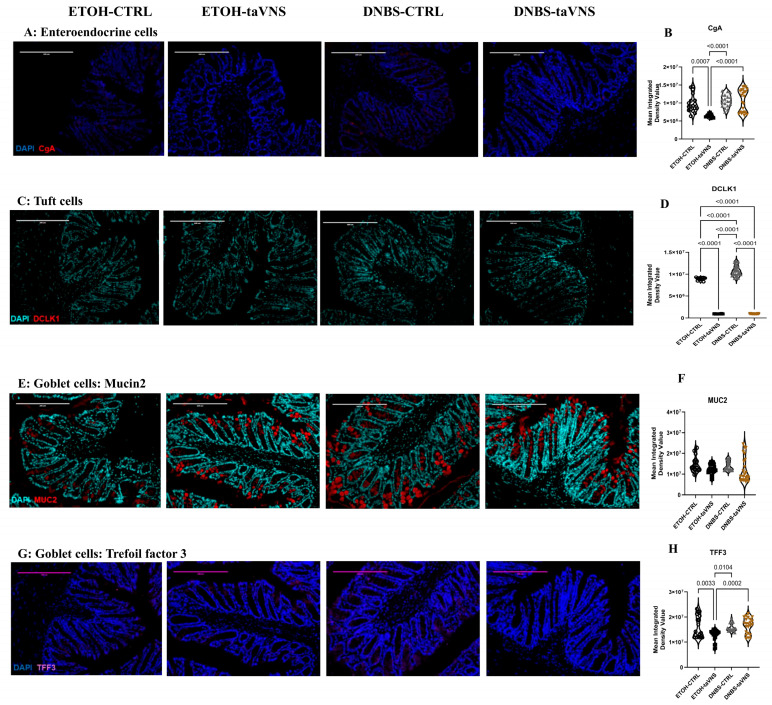
The effects of transcutaneous auricular vagus nerve stimulation (taVNS) on enteroendocrine cells, tuft cells, and goblet cells in the distal colon of a dinitrobenzene sulfonic acid (DNBS)-induced acute colitis model. The mean integrated density (MID) of the following parameters was evaluated in the distal colon using immunofluorescence (IF) staining: the enteroendocrine-cell-related chromogranin A (CgA) (**A**,**B**), tuft cell-associated doublecortin-like kinase 1 (DCLK1) (**C**,**D**), goblet cell-associated markers, including mucin2 (MUC2) (**E**,**F**) and trefoil factor 3 (Tff3) (**G**,**H**). The groups were compared using one-way ANOVA followed by a multiple parametric comparison test (Tukey’s HSD post hoc). *p*-values < 0.05 were considered statistically significant. Data were presented as mean ± SD. The scale bar was 200 μm. Each group in IF experiments consisted of n = 4 mice.

The MID of CgA was significantly decreased by taVNS in control conditions, with no modifications in DNBS colitic mice (Figure 7A,B). In both control and colitic mice, taVNS decreased the MID of DCLK1 (Figure 7C,D), with no changes in its mRNA expression levels across the groups (Supplementary Figure S3A). As shown in Figure 7E,F and Figure S3B, taVNS did not affect the MID or mRNA levels of MUC2 in either control or colitic mice. In addition, the MID of TFF3 was significantly decreased by taVNS in control mice, but not in colitic mice (Figure 7G,H).

### 2.8. The Effects of taVNS on the Colonic Proliferating, Fetal-like, and Quiescent Stem Cells in the DNBS-Induced Colitis Model

**Figure 8:**
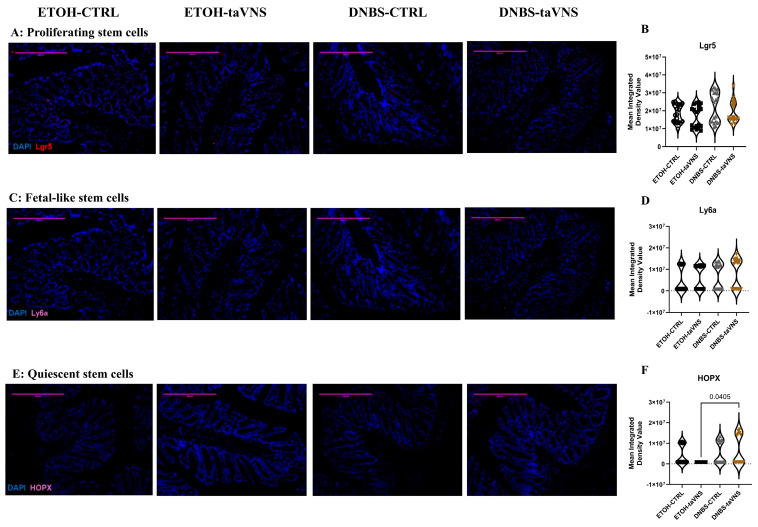
The effects of transcutaneous auricular vagus nerve stimulation (taVNS) on proliferating, fetal-like, and quiescent stem cells in the distal colon of a dinitrobenzene sulfonic acid (DNBS)-induced acute colitis model. The mean integrated density (MID) of the following parameters was evaluated in the distal colon using immunofluorescence (IF) staining: the proliferating-stem-cell-associated leucine-rich repeat-containing G-protein-coupled receptor 5 (Lgr5) (**A**,**B**), fetal-like-stem-cell-related lymphocyte antigen 6 A (Ly6A) (**C**,**D**), and quiescent stem cell marker homeodomain-only protein homeobox (HOPX) (**E**,**F**). The groups were compared using one-way ANOVA followed by a multiple parametric comparison test (Tukey’s HSD post hoc). *p*-values < 0.05 were considered statistically significant. Data were presented as mean ± SD. The scale bar was 200 μm. Each group in IF experiments consisted of n = 4 mice.

In both control and DNBS-induced colitic mice, taVNS did not affect the MID and mRNA levels of Lgr5 (Figure 8A,B and Figure S4A), Ly6a (Figure 8C,D and Figure S4B), and HOPX (Figure 8E,F and Figure S4C).

### 2.9. Correlation Between the DAI-AUC and MID of Various Epithelial Cell-Associated Markers in the DNBS-Induced Colitis Model

**Figure 9:**
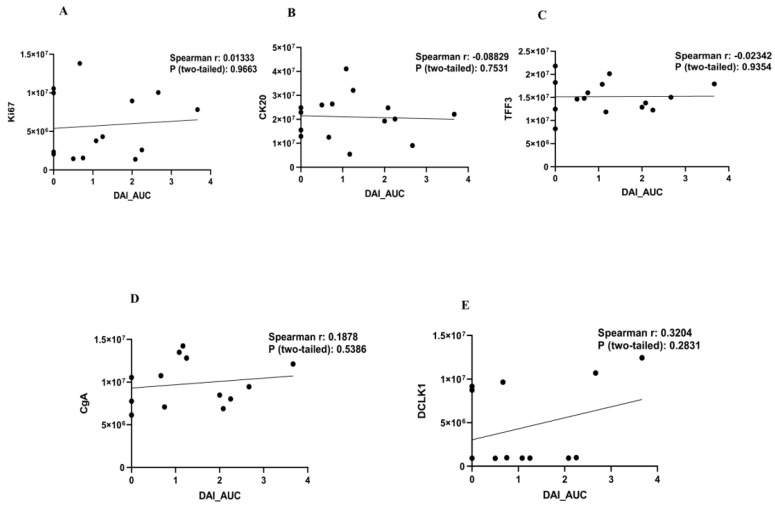
Correlation between the area under the curve (AUC) of the disease activity index (DAI) and the protein levels of differentiation and proliferation markers and gut-differentiated epithelial cell-associated markers in a dinitrobenzene sulfonic acid (DNBS)-induced acute colitis model. U-ing the data shown in Figure 6 and Figure 7 and our previously published DAI data [7], as well as the calculated AUC, the correlation between AUC-DAI and the protein levels of the following parameters was calculated for each mouse: antigen Kiel 67 (Ki67) (**A**), cytokeratin 20 (CK20) (**B**), trefoil factor 3 (TFF3) (**C**), chromogranin A (CgA) (**D**), and doublecortin-like kinase 1 (DCLK1) (**E**). Spearman’s r and corresponding *p* values were calculated using Spearman’s non-parametric test. The fitted solid line is shown for visual purposes only and was not included in the statistical analysis. Statistical significance was set at *p* < 0.05. In the correlation analysis, the data from all experimental groups were pooled, and each dot on each graph represents one mouse (3–4 mice per group, 12–16 mice total).

No significant correlation was found between the previously published DAI-AUC of the DNBS colitis model [7] and the quantified MID of Ki67 (Figure 9A), CK20 (Figure 9B), TFF3 (Figure 9C), CgA (Figure 9D), and DCLK1 (Figure 9E).

## 3. Discussion

In our previously published study, we demonstrated that taVNS has local and systemic anti-inflammatory effects on the development of acute colitis in male mice, with greater efficacy in the DSS-induced UC-like model than in the DNBS-induced CD-like model [7]. However, it remained unclear whether taVNS influenced gut epithelial barrier integrity and whether the significantly lower DAI reported in our DSS model and the partial improvement in DAI in our DNBS model [7] correlated with factors related to epithelial barrier integrity. Accordingly, the present study compared two preclinical IBD models to assess the effects of taVNS on gut barrier integrity, using markers of differentiation, proliferation, differentiated epithelial cells, and stem cells.

In homeostatic conditions, intestinal epithelial barrier integrity is maintained through balanced epithelial cell turnover and colonic stem cell proliferation and differentiation into terminally differentiated epithelial cells, including EECs, tuft cells, goblet cells, and colonocytes [23]. In colitis, however, this process is impaired, contributing to epithelial damage and a dysregulated mucosal barrier [24].

In the present study, epithelial proliferation was evaluated using Ki67 in a preventive taVNS setting. In the severe acute DSS model (5% DSS for 5 days), preventive taVNS decreased Ki67 MID in non-colitic tissues but did not modify epithelial proliferation in colitic mice. The impact of taVNS on proliferation appears context-dependent. Our Ki67 MID results in non-colitic colon are inconsistent with the increased baseline proliferation reported in direct acetylcholine receptor studies using organoids [25], likely due to taVNS’s complex regulation in vivo versus in vitro experiments. In DSS-induced colitis, intestinal proliferative activity is primarily associated with regenerative responses following DSS-induced epithelial damage [26,27]. These regenerative programs are likely activated within the epithelium in direct response to DSS rather than through taVNS. However, to test this hypothesis, the association between Ki67 and these intrinsic regenerative pathways should be assessed in future studies. Our findings differ from a previous study using a milder DSS treatment (2.5% DSS for 2 days) [28], in which Meroni et al. reported that invasive VNS decreased apoptosis and enhanced early proliferative responses. This discrepancy likely relates to differences in disease severity and timing. In the DNBS colitis model, taVNS decreased Ki67 MID in both non-colitic and colitic conditions. In non-colitic tissues exposed to the ethanol vehicle, which disrupts barrier integrity [29,30], decreased Ki67 likely links to taVNS’s control of ethanol-induced proliferation. DNBS colitis involves ethanol-induced damage to CECs, enabling DNBS access to the mucosa and protein haptenization [31]. The increased immune cell infiltration and cytokine production drive epithelial proliferation [31,32]. The Ki67 reduction in DNBS-induced colitis probably reflects taVNS’s effects on normalizing proliferative responses. These findings show taVNS effects on proliferative activity in the gut are context- and IBD-model-dependent.

Our results showed significant upregulation of CK20 by taVNS in both non-colitic and colitic conditions across DSS and DNBS models, indicating a model-independent effect on epithelial differentiation. Increased CK20 levels, even in non-colitic conditions, suggest taVNS promotes maturation of terminally differentiated epithelial cells under homeostatic conditions. The taVNS-mediated upregulation of CK20 in DSS- and DNBS-induced colitic conditions likely aims to restore epithelial differentiation balance and accelerate repair despite chemical-induced epithelial loss and mucosal injury [31,33]. A uniform CK20 increase with taVNS across models indicates a model-independent pro-differentiation effect on epithelial cells while improving epithelial integrity in homeostatic settings. As the first study evaluating taVNS effects on intestinal integrity, future research is needed to elucidate underlying mechanisms.

CgA, an EEC-related marker, is often upregulated in active colitis [34], suggesting inflammation-driven EEC activation. However, changes in CgA expression may depend on the injury context, reflecting distinct biological processes. For example, increased CgA may reflect activation of surviving EECs during inflammation [34], whereas decreased CgA may instead result from the loss of these cells following direct epithelial injury. The decrease in CgA MID in non-colitic conditions following taVNS likely reflects its homeostatic effect of inhibiting excessive EEC activation in the absence of inflammation. Our DSS model showed a significant decrease in CgA MID in DSS-CTRL groups, while maintaining it in DSS-taVNS groups, with no changes in colitic DNBS mice. The reduction in CgA MID in DSS-CTRL mice likely results from severe epithelial injury, in which DSS toxicity leads to loss of differentiated epithelial cells, including EECs [33,35]. The maintenance of CgA expression in DSS-taVNS mice supports the possibility that taVNS preserves the EEC lineage during inflammation rather than reducing it. Thus, taVNS regulates CgA expression by preserving CgA and EEC integrity during DSS-induced injury while inhibiting unnecessary EEC activation in non-colitic conditions. While CgA is known for pro-inflammatory functions in colitis, some CgA-derived peptides may have protective effects [34]. Given CgA’s complexity and vagal fiber-EEC interactions via neuropod-like structures [36,37], future studies must investigate the molecular pathways of taVNS’s effects on CgA production.

Tuft cells are another subset of differentiated CECs evaluated in this study. While these cells may interact with vagal fibres via prostaglandins, leukotrienes, and acetylcholine, no direct synaptic connections have been shown [38]. Tuft cells mediate IL-25/ILC2 immune responses in inflammation or helminth infections, while promoting barrier repair and mucus production [38,39]. Although colonic epithelium-specific depletion of DCLK1, which marks tuft cells, worsens DSS colitis [40], tuft cell hyperactivation can exacerbate chronic colitis by inducing type 2 immune responses and fibrosis, impairing tissue regeneration [39]. In the DSS model, taVNS decreased DCLK1 expression under non-colitic conditions, while in the DNBS model, this marker decreased following taVNS in both non-colitic and colitic tissues, confirming model-dependent effects. The decreased DCLK1 expression under non-colitic conditions reflects taVNS’ homeostatic functions, maintaining balance in tuft cell activation to prevent unnecessary Th2-skewed immune responses. Vagotomy studies showing reduced tuft cells highlight the VN’s role in maintaining steady states [41]. The decrease in DCLK1 expression in colitic DNBS conditions following taVNS may reflect control of pathological hyperactivity of tuft cells and the normalization of their function, consistent with VN’s role in maintaining epithelial integrity in IBD-like conditions [39].

We evaluated the effects of taVNS on goblet cell-associated molecules MUC2 and TFF3 in the DSS and DNBS models. MUC2 and TFF3 are co-expressed in goblet cells, with distinct yet complementary functions [42]. MUC2 is the main gel-like mucin from goblet cells, forming a thick inner mucus layer between the lumen and epithelial cells [43]. TFF3 is a trefoil factor peptide that promotes epithelial restitution and wound healing in IBD [44]. We observed MUC2 upregulation in DSS colitis following taVNS, but not in DNBS mice. TFF3 remained unchanged by taVNS in DSS mice but decreased in non-colitic mice of the DNBS model. These findings show context- and model-dependent effects of taVNS on goblet cell function. In the DSS model, characterized by epithelial injury and mucosal barrier disruption [33], increased MUC2 suggests taVNS pro-repair effects on mucin production through enhanced goblet cell differentiation and restoration. DNBS induces immune cell-mediated inflammation and epithelial injury [31], potentially increasing epithelial sensitivity from 30% ethanol treatment even under non-colitic conditions [29]. As TFF3 expression is regulated by cytokines and linked to epithelial restitution [44], reduced TFF3 with taVNS in DNBS non-colitic mice may indicate control of subclinical and vehicle-mediated epithelial stress. This suggests taVNS maintains TFF3 expression in the resting state under non-colitic conditions. These observations demonstrate model-specific effects of taVNS: in DSS colitis, it restores mucosal barrier integrity through MUC2, while in non-colitic DNBS conditions, it regulates epithelial restitution via TFF3.

We evaluated how taVNS modified the intestinal stem cell pool and differentiated CECs. Results on Lgr5^+^ stem cells, a multipotent subset generating all differentiated CECs [4], again confirmed taVNS’s context- and model-dependent protective effects on intestinal integrity. In non-colitic conditions, taVNS exerted a homeostatic effect, decreasing Lgr5 MID and mRNA levels. With stable epithelial turnover in non-colitic conditions, there is probably less need for extensive Lgr5^+^ stem cell activation [45], likely leading to their homeostatic downregulation by taVNS. In the DSS model under colitic conditions, Lgr5 MID and mRNA levels decreased significantly, but taVNS prevented this decrease at the mRNA level in the DSS-taVNS group. Lgr5^+^ stem cells disappear quickly upon tissue injury or inflammation [45], consistent with our Lgr5 mRNA and MID data in DSS-colitic mice. Differences in taVNS effects in non-colitic and colitic conditions in the DSS model show context-dependent modulation of Lgr5^+^ stem cells by taVNS. In the DNBS model, taVNS did not modify Lgr5^+^ stem cells in either non-colitic or colitic mice, showing model-dependent effects. These findings suggest that taVNS acts primarily as a homeostatic modulator of Lgr5^+^ stem cell dynamics rather than simply promoting epithelial repair. It reduces unnecessary Lgr5^+^ stem cell activity when epithelial turnover is balanced under physiological conditions, while preserving the Lgr5^+^ stem cell pool in DSS-induced epithelial injury. This suggests that taVNS promotes regenerative capacity primarily when epithelial integrity is compromised. The absence of similar effects in the DNBS model further indicates that the homeostatic regulatory effects of taVNS may depend on the primary mechanism of injury and are more evident in the DSS model, which is characterized by direct epithelial damage. We evaluated whether taVNS affects HOPX^+^ quiescent and reserve intestinal stem cells, which serve as backup during severe injury and Lgr5^+^ stem cell loss [46,47]. It decreased HOPX MID in non-colitic mice in the DSS model, reflecting homeostatic effects on intestinal barrier integrity. Despite decreased HOPX MID and mRNA levels in DSS colitic conditions, taVNS did not affect these cells in either the DNBS model or DSS colitic mice. These differences between models regarding HOPX^+^ stem cells align with taVNS’s model-dependent nature. When examining Ly6a^+^ intestinal stem cells, which are fetal-like, injury-induced regenerative cells [26], we observed no significant changes in Ly6a mRNA expression or MID in either colitis model. Although Ly6a^+^ stem cells are typically upregulated following epithelial injury [26], preventive taVNS did not alter this stem cell population, likely because Ly6a expression is transient, and any changes may have occurred outside the tissue collection time points examined in the present study [48].

We also evaluated relationships between our published DAI data [7] and the MID of epithelial-related markers in DSS and DNBS models. While we found no significant correlation between DAI-AUC in DSS and DNBS mice and the MID of Ki67, CK20, TFF3, MUC2, and DCLK1, we observed a significant negative correlation between DAI-AUC and CgA MID in the DSS colitis model. Given this finding and the previously discussed context-dependent effects of taVNS on CgA MID in DSS and DNBS models, CgA may be considered an indicator of EEC integrity rather than inflammatory activation alone, at least in the DSS colitis model, where DAI is associated with direct epithelial injury [33], including EECs, rather than immune-mediated inflammation seen in the DNBS model [31]. The negative CgA-DAI correlation in our DSS model suggests taVNS reduces DAI by preserving EEC integrity. Future studies should examine direct interactions between vagal fibers and EECs and how these cells contribute to reducing DSS-induced colitis severity. This could be tested by inducing DSS colitis with taVNS stimulation in EEC-specific CgA-conditional knockout mouse models to determine whether taVNS effects are altered in the absence of CgA.

To further evaluate the effects of taVNS on intestinal barrier function, we investigated TEER, paracellular permeability, and epithelial transport activity in response to various drugs in the distal and proximal colon of the DSS model. Besides the distal colon, the main site of DSS-induced tissue injury [33], we included the proximal colon for its importance in water and electrolyte reabsorption, distinct ion transport [49], and denser VN innervation [14]. Our results showed an increase in TEER, an electrophysiological measure of epithelial barrier integrity linked to tight junctions [50], in distal non-colitic and proximal colitic colon tissues, suggesting region- and context-dependent taVNS protective effects on TEER and, likely, on its associated tight junction organization. Previous mouse studies have shown higher TEER in the proximal colon than in the distal colon under steady-state conditions, suggesting greater barrier integrity in the proximal colon [51]. However, in DSS colitis, DSS decreases TEER regionally, with the proximal colon showing higher sensitivity to DSS-induced injury during early colitis phases [51]. To confirm the region-dependent effects of taVNS, future taVNS studies are needed to evaluate changes in tight junction molecules in the distal and proximal colon. Recent studies have further highlighted intestinal barrier restoration through coordinated regulation of tight junction proteins and MUC2, mediated by inflammatory signaling pathways such as nuclear factor kappa light-chain enhancer of activated B cells (NF-κB) in UC models [52]. Similar mechanisms of epithelial injury and repair are also observed in other mucosal tissues, such as lungs, where inflammation-driven signaling pathways such as Toll-like receptor 4/NF-κB and oxidative stress contribute to barrier impairment [53], highlighting conserved pathways of epithelial damage across mucosal tissues. This suggests potential barrier-modulatory effects of taVNS that warrant further investigation in future studies. When examining paracellular permeability using FITC-dextran flux, we found no significant effect of taVNS in the proximal colon. In the distal colon of colitic mice, taVNS reduced FITC-dextran flux, which approached statistical significance at 120 min, suggesting a trend toward decreased paracellular permeability. However, this effect did not reach statistical significance and should therefore be interpreted with caution. Previous DSS colitis studies have shown a regional increase in paracellular FITC-dextran flux, especially in the proximal colon [51]. Preclinical and human studies using VNS or taVNS have shown barrier function improvement by regulating tight junction proteins and controlling stress-induced paracellular permeability [54,55]. Given this, our findings suggest that taVNS modifies TEER and paracellular permeability in a region- and context-dependent manner. Our results showed no significant changes in taVNS effects on distal ISC or epithelial transport activity. However, taVNS enhanced drug-induced ISCs in the proximal non-colitic colon in response to amiloride, IBMX, forskolin, carbachol, and bumetanide. The effects of taVNS on active ion transport pathways, such as ENaC-sensitive Na^+^ absorption, Ca^2+^-dependent secretion, NKCC-dependent Cl^−^ uptake, and CFTR/cAMP-dependent secretion [50], are more evident in the proximal colon, which can be attributed to denser VN innervation [14] or higher ion transport capacity due to less DSS-induced structural damage [33]. Overall, based on previous Ussing-related analyses [51], our findings show that taVNS regulates epithelial ion transport and barrier integrity independently, with a region- and context-dependent function.

We acknowledge some limitations of this study. To avoid estrous cycle-related variations and establish our preventive models and stimulation conditions, we used only male mice in our experiments. Given that previous studies have shown sex-related differences in epithelial barrier integrity [56,57,58], DAI [59], and VN activity and morphology [60,61,62], we acknowledge that this approach limits the generalizability of our findings to females. Human studies have shown that females generally exhibit higher baseline vagus nerve activity, which is likely related to sex hormone receptors expressed in the brainstem that mediate the reception of peripheral signals [60,61,62]. Therefore, given the effects of female sex hormones on vagal tone, future studies are needed to evaluate these findings in female mice using the same stimulation parameters.

In our daily 10-min taVNS or sham stimulation protocol, mice were anesthetized with 2% isoflurane throughout the experiment because of its ease of use and controllability in mouse studies. Several studies have reported that isoflurane suppresses vagus nerve activity in a dose-dependent manner [63]. Moreover, isoflurane may directly or indirectly affect intestinal epithelial barrier integrity by decreasing epithelial apoptosis and inflammatory responses, thereby promoting barrier integrity following injury [64,65]. Although both the taVNS and sham groups were exposed to identical anesthesia conditions, the potential confounding effects of isoflurane cannot be excluded. Specifically, anesthesia may reduce baseline vagus nerve activity, thereby masking the full efficacy of taVNS, particularly when subtle biological effects are expected. Therefore, the use of isoflurane may have reduced the present study’s ability to detect the full effects of taVNS on intestinal epithelial integrity.

Both the DSS- and DNBS-induced colitis models used in this study were preventive. Therefore, future research should evaluate the effects of taVNS, using the same stimulation parameters, in established (therapeutic) IBD models to determine whether it can enhance intestinal barrier integrity and preserve intestinal stem cells and differentiated epithelial cells after disease onset. In addition, to evaluate the potential effectiveness of taVNS on mucosal healing and epithelial repair, future studies should use chronic IBD models and multiple DSS and DNBS treatment cycles with recovery periods.

In several experiments, the sample size was relatively small, potentially limiting the study’s ability to detect subtle differences between the groups, especially for our secondary endpoints. Additionally, given the evaluation of multiple epithelial and stem cell markers, some findings should be interpreted cautiously and verified in future research with larger sample sizes.

The primary findings of this study were based on the distal colon, which is considered the main target of DSS- and DNBS-induced localized epithelial damage [31,66]. However, it has been demonstrated that different regions of the mouse colon exhibit distinct VN innervation densities, with the proximal colon having the highest innervation and the distal colon the lowest [14]. Additionally, our Ussing chamber data revealed distinct taVNS responses in the distal and proximal colon. Therefore, it is strongly recommended that future studies evaluate the potential region-dependent effects of taVNS on epithelial barrier integrity.

## 4. Materials and Methods

### 4.1. Mice

Following the ethics-reduction approach, the experiments were conducted with the same mouse cohort previously documented by our laboratory [7]. Male C57BL/6 mice (11–12 weeks old, 22–28 g) were purchased from Central Animal Care Services (CACS) (Bannatyne Campus, University of Manitoba) or Charles River (Sherbrook, QC, Canada). The cages contained two mice each and were kept in a pathogen-free room at the Animal Care Facility of the University of Manitoba. Based on the pre-developed exclusion criteria, mice losing 20% of their body weight were humanely euthanized by 2-min anesthesia (4% isoflurane and 1 L/min oxygen flow rate), followed by cervical dislocation. All the experiments were approved by the University of Manitoba Animal Ethics Committee (Protocol Numbers: 20-064 and 25-001).

### 4.2. TaVNS Stimulation in the DSS-Induced Acute Colitis Model

Sixteen male C57BL/6 mice were randomly assigned to four groups: DSS-taVNS, DSS-control (CTRL), CTRL-taVNS, and CTRL-CTRL, with four mice per group. The preventive taVNS and control (no stimulation) procedures were initiated 24 h before DSS-induced colitis. For the taVNS group, after anesthesia (4% isoflurane and 1 L/min oxygen flow), the mice received stimulation through the auricular branch of the VN innervating the cymba concha of both ears using an auricular nerve stimulator (Vagustim Animal Research Device (ARD), Vagustim, Istanbul, Turkey) with the following parameters: frequency, 20 Hz; voltage, 10 V; pulse width, 500 µs; on/off duration, 30 s; and total stimulation time, 10 min. During stimulation, the mice were maintained anesthetized using 2% isoflurane and an oxygen flow of 0.8 L/min. In the control (sham stimulation) group, the same procedure was followed, except for the actual stimulation. Both conditions were maintained daily throughout the experiment. To induce acute colitis in the DSS-CTRL and DSS-taVNS groups, 5% DSS (wt/vol) (molecular weight, 40 kDa; Thermo Fisher Scientific, Mississauga, ON, Canada) was added daily to their drinking water for 120 h. Meanwhile, the CTRL-CTRL and CTRL-taVNS mice received regular water during the same period. Throughout the experiment, the disease activity index (DAI), a composite measurement of weight loss percentage, fecal bleeding, and stool consistency, was assessed daily [7,67]. After euthanizing the mice on the day of sacrifice (144 h relative to the start of taVNS/sham stimulation) through a 2-min anesthesia (4% isoflurane and 1 L/min oxygen flow rate), followed by cervical dislocation, the collected colon samples were used for further experiments. Except for stimulation conditions and DAI evaluation, all other data acquisition and experiments were conducted without knowledge of the groups. All mouse-handling procedures were performed by the same person under the same conditions to minimize potential variables.

### 4.3. TaVNS Stimulation in the DNBS-Induced Colitis Model

Based on receiving DNBS/ethanol treatment and taVNS/no stimulation, sixteen male C57BL/6 mice were randomly divided into four groups: DNBS-taVNS, DNBS-CTRL, ETOH-taVNS, and ETOH-CTRL. The taVNS groups received 10 min of taVNS with the same stimulation parameters and anesthesia process as the DSS model, 24 h before receiving DNBS/ethanol treatments (preventive model), and this process continued until the end of the experiment. Meanwhile, the CTRL mice received no stimulation. After 24 h, depending on their assigned group, the mice received 100 µL of 30% ethanol or 4 mg of DNBS dissolved in 30% ethanol via intrarectal administration. PE-90 tubing (10 cm length, ClayAdam, Parsippany, NJ, USA) was used for injections. During the procedure, the mice were anesthetized (2% isoflurane and an oxygen flow of 0.8 L/min), and the tube connected to a tuberculin syringe (BD, Mississauga, ON, Canada) was inserted 3.5 cm into the colon. The DAI was recorded daily during the experiment. On the final day (120 h relative to the start of taVNS/sham stimulation), the mice were euthanized using the same anesthesia-cervical dislocation process, and their distal colon tissues were collected for further analysis. Similar to the DSS model, all mouse-handling experiments were conducted by the same person under the same conditions.

### 4.4. Immunofluorescence (IF) Staining

The distal colon tissues were used to assess the expression of markers related to different gut cell states: proliferating (Lgr5) (Bio-Techne, Minneapolis, MN, USA), fetal-like (Ly6A) (Santa Cruz, San Diego, CA, USA), and quiescent (HOPX) stem cells (Santa Cruz, San Diego, CA, USA); differentiation marker cytokeratin 20 (CK20) (Cell Signaling Technology, Danvers, MA, USA) and the proliferation marker antigen Kiel 67 (Ki67) (Invitrogen, Mississauga, ON, Canada); and gut epithelial cell markers such as tuft cell-associated doublecortin-like kinase 1 (DCLK1) (Santa Cruz, San Diego, CA, USA), goblet cell-associated mucin2 (MUC2) (Abcam, Inc Waltham, MA, USA), trefoil factor 3 (TFF3) (Santa Cruz, San Diego, CA, USA), and Chromogranin A (CgA) (Invitrogen, Mississauga, ON, Canada).

To prepare tissues for staining, they were deparaffinized by washing three times with xylene, rehydrated with 100%, 95%, and 70% ethanol, and then rinsed with water. Antigen retrieval was performed by heating in sodium citrate for 15 min, followed by three phosphate-buffered saline (PBS) washes, and permeabilization with a buffer containing 0.2% gelatin and 0.25% Triton X-100 (Invitrogen, Mississauga, ON, Canada) for 10 min. The samples were blocked in a buffer with 0.2% gelatin, 0.25% Triton X-100, and 5% bovine serum albumin (BSA) (Invitrogen, Mississauga, ON, Canada) for 3 h, and then incubated overnight at 4 °C with primary antibodies. After washing, the tissues were permeabilized again, and then incubated with fluorescent secondary antibodies (Invitrogen, Mississauga, ON, Canada) for 1 h at room temperature, followed by DAPI (Sigma-Aldrich, Mississauga, ON, Canada) staining for 10 min. The slides were finally examined under an Evos imaging microscope (Thermo Fisher Scientific, Mississauga, ON, Canada). The MID for each marker was measured across the entire microscopic field of view using Fiji 2.16.0 ImageJ software, without manually selecting specific regions of interest. Each marker was analyzed from four mice per group, with one section taken from each mouse, resulting in four sections per group. For every section, 6–10 microscopic fields were imaged and measured. Consistent acquisition and analysis settings, such as exposure time, were applied across all markers and experimental groups.

### 4.5. Ex Vivo Intestinal Permeability: Ussing Chamber and FITC-Dextran

Proximal and distal colon samples were sliced lengthwise, washed gently in Krebs buffer with 10 mM glucose, placed in tissue holders, and positioned between compartments of a voltage-clamp device (VCC MC8; Physiologic Instruments, San Diego, CA, USA). Each chamber was filled with a Krebs/glucose solution and oxygenated with carbogen (95% O2 and 5% CO_2_) throughout data collection. Transepithelial electrical resistance (TEER) was measured using Acquire and Analyze software version 2.3 (Physiologic Instruments, San Diego, CA, USA). To assess the epithelial transport activity in response to various drugs and as presented as short-circuit current (ISC), drugs such as Indomethacin (Sigma-Aldrich, St. Louis, MO, USA) were added to both chambers, Amiloride (Sigma-Aldrich, St. Louis, MO, USA) to the apical chamber, 3-isobutyl-1-methylxanthine (IBMX) and Forskolin (both Sigma-Aldrich, St. Louis, MO, USA) to both chambers, and Carbachol and Bumetanide (Sigma-Aldrich, MO, USA) to the serosal chamber at final concentrations of 10 μM. FITC-dextran (4 kDa, Sigma-Aldrich, MO, USA) was added to the apical chamber at 100 μg/mL, and samples from the serosal chamber were collected every 30 min for 2 h to evaluate paracellular permeability [68].

### 4.6. Quantitative Real-Time Reverse-Transcription Polymerase Chain Reaction (qRT-PCR)

Distal colon tissues were minced and homogenized using an ultrasonic processor (PRO Scientific Inc., Oxford, CT, USA) in 1 mL of TRIzol™ (Invitrogen, ThermoFisher Scientific, Waltham, MA, USA). Total RNA was extracted and purified using an RNA kit (Life Technologies, Grand Island, NY, USA). The RNA was reverse transcribed into cDNA using SuperScript VILO cDNA Synthesis Master Mix (Invitrogen, Grand Island, NY, USA). The cDNA was used for gene expression analysis by qRT-PCR with Power SYBR Green Master Mix (Life Technologies, Burlington, ON, Canada) on a Roche LightCycler 96. Expression levels of target genes, normalized to the housekeeping gene TATA-box binding protein (*TBP*), were calculated using the Roche software version 1.1 [69]. The list of genes and primers used is provided in Table 1.

### 4.7. Data Analysis

Statistical differences among the groups were evaluated in GraphPad Prism (version 9; GraphPad Software, Inc., La Jolla, CA, USA) using one-way or two-way ANOVA, followed by a multiple parametric comparison test, Tukey’s HSD post hoc, depending on the number of variables studied. For a single variable, such as treatment groups, one-way ANOVA was used. When there was more than one independent factor, two-way ANOVA was applied to assess interactions between variables. The main effects of each factor and their interaction were evaluated. To evaluate the correlation between the area under the curve (AUC) of our previously published DAI in both DSS- and DNBS-induced acute colitis models [7] and the MID of various markers measured by IF staining, Spearman’s nonparametric test was used. The sample size of four mice per group was selected based on previous studies from our laboratory, which showed that the sample size was sufficient to detect significant differences in the primary outcome measures. Therefore, the same sample size was used in the present study. All generated data were included in the analysis. Two-tailed *p*-values below 0.05 were considered statistically significant. The data are presented as the mean ± SD.

## 5. Conclusions

In summary, our findings showed that the preventive effects of taVNS on intestinal barrier integrity and epithelial ion transport activity are model-, context-, and region-dependent. Although taVNS increased intestinal cell differentiation in both UC- and CD-like colitis models, its effects on stem cells and differentiated CECs were model-dependent. This should be considered in future studies focusing on the preventive effects of taVNS on IBD. We also showed that taVNS may modify CEC differentiation, favoring epithelial lineages such as EECs in DSS colitis. EECs strongly negatively correlated with the DAI in the DSS model, suggesting a link between these cells and taVNS’s preventive effects on the DAI. In non-colitic conditions, taVNS downregulated colonic proliferative activity and stem cell pools, as well as some specific differentiated CECs, indicating the context-dependent nature of taVNS. To better understand the effects of taVNS on CECs and stem cells, future functional studies should evaluate potential modifications in the molecular pathways related to taVNS or its mediators in each subset of CECs and stem cells. Moreover, future research should evaluate the therapeutic potential of taVNS in established colitis models.

## Figures and Tables

**Table 1:** Primers.

Gene Name	Forward (5′-3′)	Reverse (5′-3′)
*Dclk1*	CTGGGTTAATGATGATGGTCTCC	ACAGAAACTCCTGCTGCAGT
*Muc2*	GATGGCACCTACCTCGTTGT	GTCCTGGCACTT GTTGGAAT
*Lgr5*	CTTCCGAATCGTCGATCTTC	AACGATCGCTCTCAGGCTAA
*Ly6a*	AGGAGGCAGCAGTTATTGTGG	CGTTGACCTTAGTACCCAGGA
*Hopx*	TCTCCATCCTTAGTCAGACGC	GGGTGCTTGTTGACCTTGTT
*TBP*	CGTGAATCTTGGCTGTAAACT	GTCCGTGGCTCTCTTATTCT

*Dclk1*: Doublecortin-like kinase, *Muc2*: Mucin2, *Lgr5*: Leucine-rich repeat-containing G-protein coupled receptor 5, *Ly6a*: Lymphocyte antigen 6 A, *Hopx*: Homeodomain-only protein homeobox, *TBP*: TATA-box binding protein.

## Data Availability

The raw data supporting the conclusions of this article will be made available by the authors on request.

## Supplementary Materials

The following supporting information can be downloaded at: https://www.mdpi.com/article/10.3390/ijms27146109/s1.

## Abbreviations

ARD	Animal research device
AUC	Area under the curve
BSA	Bovine serum albumin
CACS	Central animal care services
CD	Crohn’s disease
CEC	Colonic epithelial cell
CgA	Chromogranin A
CK20	Cytokeratin 20
CTRL	Control
DAI	Disease activity index
DCLK1	Doublecortin-like kinase 1
DMV	Dorsal motor nucleus of the VN
DNBS	Dinitrobenzene sulfonic acid
DSS	Dextran sulfate sodium
EEC	Enteroendocrine cell
GI	Gastrointestinal
HOPX	Homeodomain-only protein homeobox
IBD	Inflammatory bowel disease
IBMX	3-isobutyl-1-methylxanthine
IF	Immunofluorescence
ISC	Short-circuit current
Ki67	Antigen Kiel 67
Lgr5	Leucine-rich repeat-containing G protein-coupled receptor 5
Ly6A	Lymphocyte antigen 6 A
MID	Mean integrated density
MUC2	Mucin2
NTS	Nucleus tractus solitarii
PaNS	Parasympathetic nervous system
PBS	Phosphate-buffered saline
qRT-PCR	Quantitative real-time reverse-transcription polymerase chain reaction
taVNS	Transcutaneous auricular vagus nerve stimulation
TBP	TATA-box binding protein
TEER	Transepithelial electrical resistance
TFF3	Trefoil factor 3
UC	Ulcerative colitis
VN	Vagus nerve
VNS	Vagus nerve stimulation
