# Supplementary material for: Context-Dependent Modulation of Epithelial Barrier Integrity and Intestinal Permeability by Transcutaneous Auricular Vagus Nerve Stimulation in Two Preclinical Models Mimicking Ulcerative Colitis and Crohn’s Disease: A Descriptive Analysis
Source: Int J Mol Sci. 2026 Jul 8;27(14):6109. doi: 10.3390/ijms27146109 (PMC13410186; doi:10.3390/ijms27146109)
Supplement: Supplementary file 1 [file ijms-27-06109-s001.zip › ijms-4371361-supplementary.pdf]

A

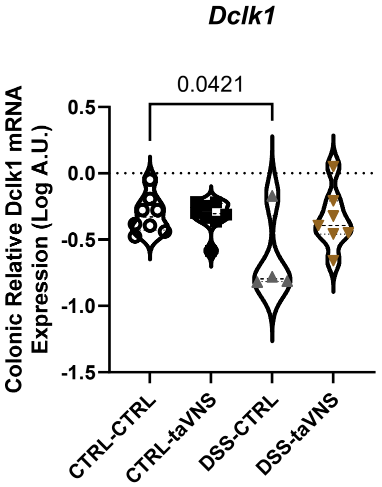

B

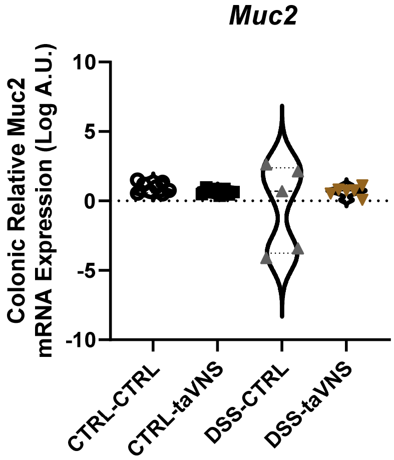

Figure S1. The effects of transcutaneous auricular vagus nerve stimulation (taVNS) on mRNA expression levels of tuft cell-associated doublecortin-like kinase 1 (*Dclk1*) and goblet cell-associated markers, including mucin2 (*Muc2*) in the distal colon of a dextran sulfate sodium (DSS)-induced acute colitis model.

The taVNS and sham (control) groups were subjected to either taVNS stimulation (20 Hz frequency, 500 µs pulse width, 10-minute duration with 30-second on/off intervals) or sham stimulation (anesthesia without electrical stimulation), respectively, beginning 24 hours before the induction of acute colitis. This procedure was repeated until the end of the experiment. Twenty-four hours later, 5% DSS was added to the drinking water of the DSS groups for 120 hours, whereas the control (non-colitic) groups received regular drinking water. The mRNA levels of *Dclk1* **(A)** and *Muc2* **(B)** were evaluated in the distal colon using qRT-PCR. The groups were statistically compared using one-way ANOVA, followed by a multiple parametric comparison test (Tukey's HSD post hoc). P-values < 0.05 were considered statistically significant. Data were presented as mean ± SD. Each group consisted of n = 4-8 mice.

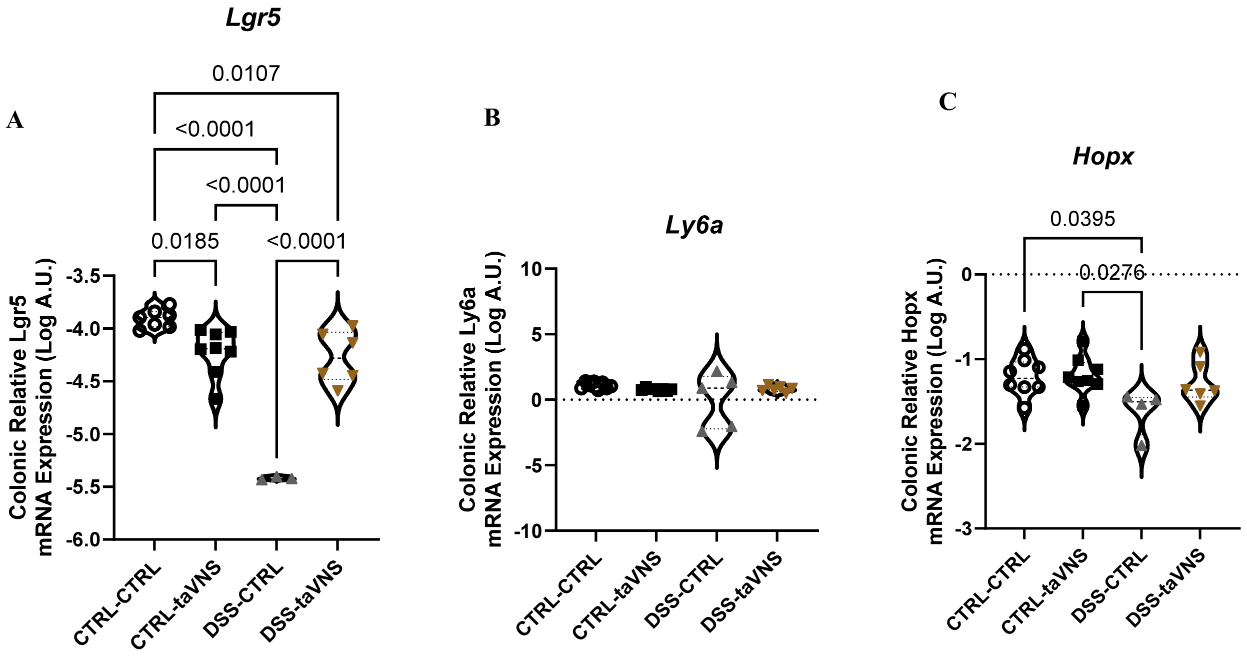

Figure S2. The effects of transcutaneous auricular vagus nerve stimulation (taVNS) on mRNA expression levels of proliferating-stem-cell-associated leucine-rich repeat-containing G-protein-coupled receptor 5 (*Lgr5*), fetal-like-stem-cell-related lymphocyte antigen 6 A (*Ly6A*), and quiescent stem cell marker homeodomain-only protein homeobox (*HOPX*) in the distal colon of a dextran sulfate sodium (DSS)-induced acute colitis model.

A procedure similar to that described in the caption of Figure Supp 1 was followed. The mRNA levels of *Lgr5* **(A)**, *Ly6a* **(B)**, and *Hopx* **(C)** were evaluated in the distal colon using qRT-PCR. The groups were statistically compared using one-way ANOVA, followed by a multiple parametric comparison test (Tukey's HSD post hoc). P-values < 0.05 were considered statistically significant. Data were presented as mean ± SD. Each group consisted of n = 3-8 mice.

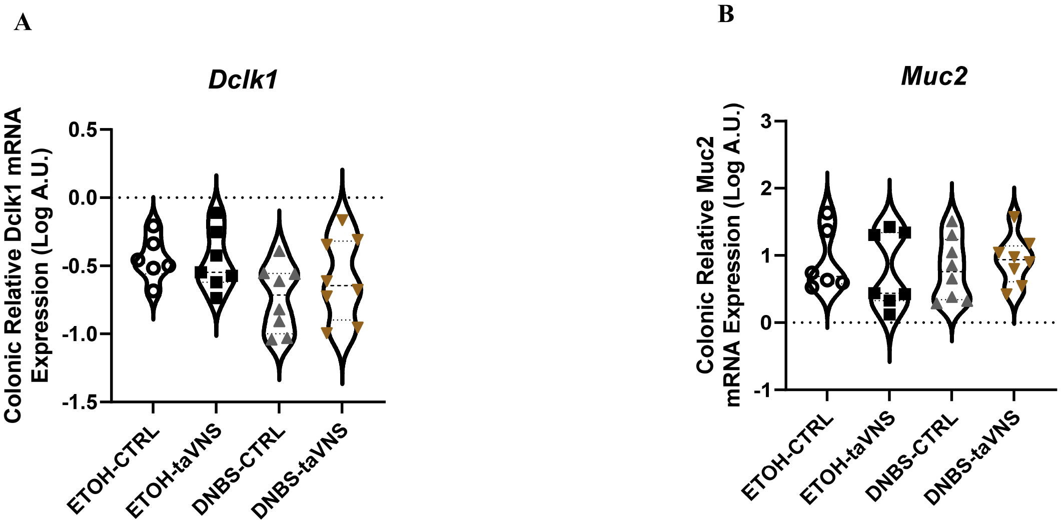

Figure S3. The effects of transcutaneous auricular vagus nerve stimulation (taVNS) on mRNA expression levels of tuft cell-associated doublecortin-like kinase 1 (*Dclk1*) and goblet cell-associated markers, including mucin2 (*Muc2*) in the distal colon of a dinitrobenzene sulfonic acid (DNBS)-induced acute colitis model.

The taVNS and sham (control) groups were subjected to either taVNS stimulation (20 Hz frequency, 500 µs pulse width, 10-minute duration with 30-second on/off intervals) or sham stimulation (anesthesia without electrical stimulation), respectively, beginning 24 hours before the induction of acute colitis. This procedure was repeated until the end of the experiment. Twenty-four hours later, the DNBS groups received a single intrarectal injection of 100 µL containing 4 mg DNBS per mouse dissolved in 30% ethanol (ETOH), whereas the ETOH control groups received 100 µL of 30% ethanol alone. The mRNA levels of *Dclk1* **(A)** and *Muc2* **(B)** were evaluated in the distal colon using qRT-PCR. The groups were statistically compared using one-way ANOVA, followed by a multiple parametric comparison test (Tukey's HSD post hoc). P-values < 0.05 were considered statistically significant. Data were presented as mean ± SD. Each group consisted of n = 5-8 mice.

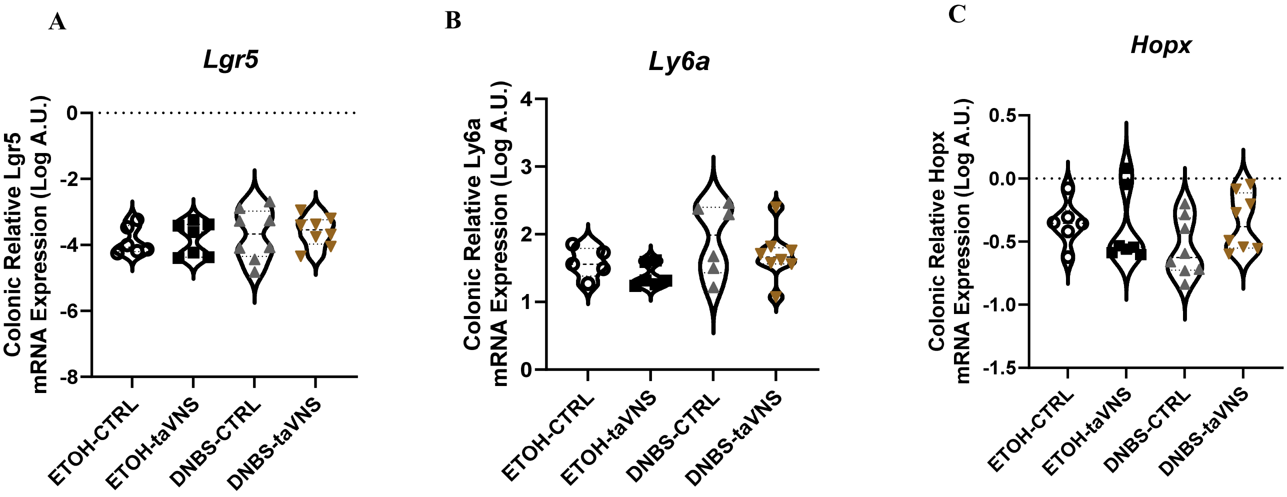

Figure S4. The effects of transcutaneous auricular vagus nerve stimulation (taVNS) on mRNA expression levels of proliferating-stem-cell-associated leucine-rich repeat-containing G-protein-coupled receptor 5 (*Lgr5*), fetal-like-stem-cell-related lymphocyte antigen 6 A (*Ly6A*), and quiescent stem cell marker homeodomain-only protein homeobox (*HOPX*) in the distal colon of a dinitrobenzene sulfonic acid (DNBS)-induced acute colitis model.

A procedure similar to that described in the caption of Figure Supp 3 was followed. The mRNA levels of *Lgr5* **(A)**, *Ly6a* **(B)**, and *Hopx* **(C)** were evaluated in the distal colon using qRT-PCR. The groups were statistically compared using one-way ANOVA, followed by a multiple parametric comparison test (Tukey's HSD post hoc). P-values < 0.05 were considered statistically significant. Data were presented as mean $\pm$ SD. Each group consisted of $n = 5$-8 mice.
